# *Mycobacterium intracellulare* ‐Induced Flexor Tenosynovitis of the Forearm

**DOI:** 10.1002/ccr3.71698

**Published:** 2025-12-15

**Authors:** Hideharu Hagiya, Shinnosuke Fukushima, Kenta Nakamoto, Kohei Oguni

**Affiliations:** ^1^ Department of Infectious Diseases Okayama University Hospital Okayama Japan

**Keywords:** *Mycobacterium avium* complex, *Mycobacterium intracellulare*, soft tissue infection, tenosynovitis

## Abstract

We herein document a clinically challenging case of *Mycobacterium intracellulare* tenosynovitis in an immunocompromised patient with rheumatoid arthritis. Diagnosing nontuberculous mycobacterial tenosynovitis is often challenging because it often mimics rheumatoid arthritis flares. Combination antimycobacterial therapy combined with surgical intervention is essential for the treatment of this refractory condition.

## 1 Case Presentation

**FIGURE 1:**
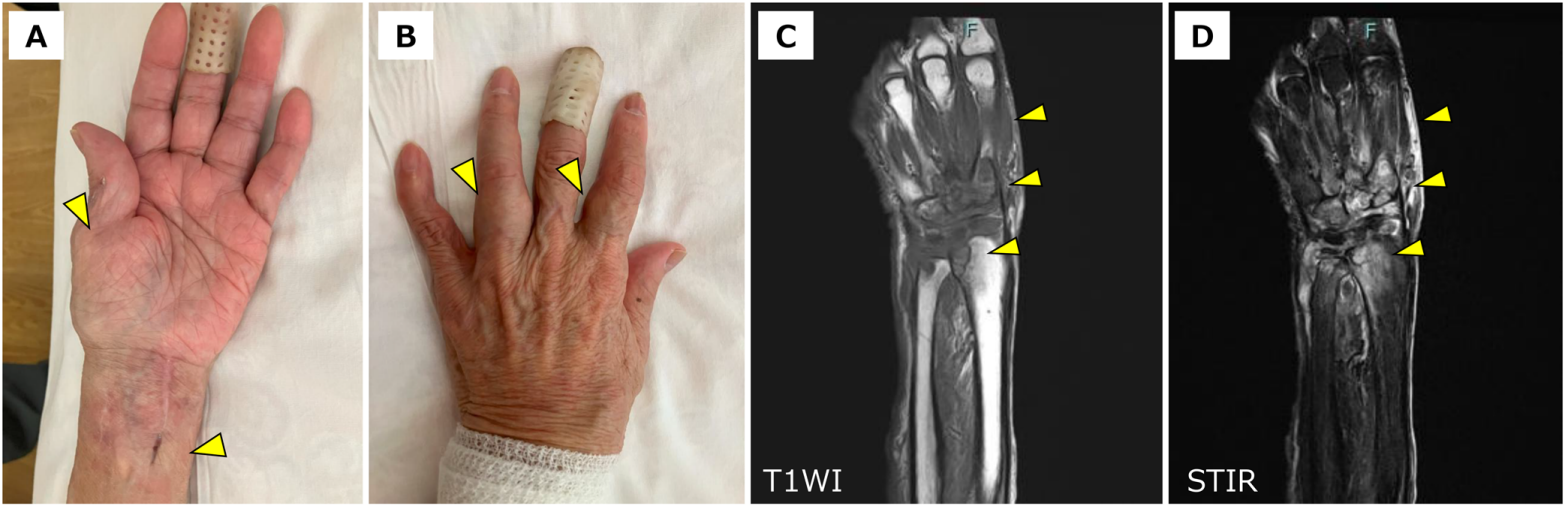
(A, B) Gross appearance of the left forearm demonstrating significant peridigital swelling. (C, D) MRI findings showing medullary osseous edema with concomitant periarticular soft tissue inflammatory changes.

A 74‐year‐old Japanese woman with a history of rheumatoid arthritis, managed over the past 4 years with various biological agents including methotrexate, sulfasalazine, abatacept, sarilumab, and ozoralizumab, developed tenderness in her left forearm and hand that had persisted for approximately 2 years. Magnetic resonance imaging (MRI) of her left forearm revealed diffuse flexor tendinitis accompanied by bone marrow edema extending from the wrist to the metacarpal bones. She underwent flexor tenosynovectomy, and *Mycobacterium intracellulare* was isolated from the resected tissue. Under the diagnosis of nontuberculosis‐induced flexor tenosynovitis, combination therapy with azithromycin, ethambutol, and rifabutin was initiated. Due to drug‐induced fever caused by rifabutin and hepatic injury associated with azithromycin, the antimicrobial regimen was modified to dual therapy with clarithromycin and ethambutol. Subsequently, intravenous amikacin was added to the treatment but was discontinued because of dizziness and ototoxicity. Clofazimine was then administered; however, it induced QT interval prolongation. Despite treatment, inflammatory changes progressed from the forearm to the wrist joint and digits (Figure 1A,B), and magnetic resonance imaging revealed progressive bone marrow edema from the carpal to metacarpal region with surrounding soft tissue edema (Figure 1C,D). The patient underwent a second surgical intervention with tenosynovectomy of the left hand and digital joints. Although hand dysfunction persists, her overall condition has improved. The patient is currently undergoing triple therapy with clarithromycin, ethambutol, and sitafloxacin, which is planned to be continued for several years.

## 2 Discussion

Tenosynovitis caused by nontuberculous mycobacteria requires both repeated surgical debridement and prolonged multidrug antimicrobial therapy [1], underscoring the importance of accurate diagnosis. Characteristic histopathological manifestations of rheumatoid arthritis‐associated tenosynovitis encompass synovial hyperplasia with leukocytic infiltration, predominantly CD4+ T lymphocytes and CD68+ macrophages [2]. However, such pathological changes were not evident in the present case. Rice body formation, an uncommon response to synovial membrane inflammation, may manifest in chronic inflammatory conditions, including rheumatoid arthritis and tuberculosis, resulting from microinfarction of synovium and fibrinous deposition. Recent literature indicates that nontuberculous mycobacterial infections can produce such multiple granular white corpuscles at the infected sites of tenosynovitis [3]. Nevertheless, neither radiological evaluation nor intraoperative findings demonstrated evidence of rice body formation in this particular case. Due to the rarity of the disease, the distinct clinicopathological entity of nontuberculous mycobacterial tenosynovitis remains inadequately characterized.

## Author Contributions

**Hideharu Hagiya:** writing – original draft. **Shinnosuke Fukushima:** writing – review and editing. **Kenta Nakamoto:** writing – review and editing. **Kohei Oguni:** writing – review and editing.

## Consent

Written informed consent was obtained from the patient for the publication.

## Conflicts of Interest

The authors declare no conflicts of interest.

## Data Availability

The authors have nothing to report.
